# Improved survival with higher radiation dose for esophageal squamous cell carcinoma patients treated with definitive chemoradiotherapy

**DOI:** 10.18632/oncotarget.19030

**Published:** 2017-07-06

**Authors:** Yuxia Deng, Chao Bian, Hua Tao, Haijun Zhang

**Affiliations:** ^1^ Department of Oncology, Zhongda Hospital, Medical School, Southeast University, Nanjing, China; ^2^ Ningxia Medical University, Yinchuan, Ningxia, China; ^3^ Department of Radiation Oncology, Jiangsu Cancer Institute and Hospital, Nanjing, China

**Keywords:** esophageal squamous cell carcinoma, radiotherapy, overall survival, progression-free survival

## Abstract

**Purpose:**

The optimal radiation dose for patients with esophageal squamous cell carcinoma (ESCC) has long been debated. We undertook the retrospective study to evaluate the survival impact of high dose vs standard dose in patients with stage II–III esophageal cancer treated with definitive chemoradiotherapy (CRT).

**Results:**

A total of 137 patients were included in our study, 63 patients classified as standard-dose group and 74 as high-dose group. For the 63 patients in the standard-dose group, the median PFS and the 1-, 2-, and 3-year PFS rates were 12.6 months, 58.0%, 26.0% and 12.0%, respectively; for the 74 patients in the high-dose group, they were 20.0 months, 80.1%, 31.0% and 20.0%, respectively (*P =* 0.013). The median OS of the patients in the standard-dose group and high-dose group groups were 19.0 months and 26.6 months, respectively, and the 1-, 2- and 3-year survival rates were 78.0%, 39.0%, and 24.0% , and 89.0%, 61.0%, and 30.0%, respectively (*P =* 0.037). Besides the rate of grade ≥ 3 acute irradiation esophagitis in the high-dose group (10.5% versus. 2.2%, *P* < 0.01), there were no significantly differ of treatment-related toxicities between the two groups.

**Materials and Methods:**

According to the radiation dose, patients from 2010 to 2014 were allocated into either the standard-dose group (50–50.4 Gy) or the high-dose group (≥ 59.4 Gy). Overall survival (OS), progression-free survival (PFS) and treatment-related toxicities were assessed and compared between the two groups.

**Conclusions:**

Our findings suggest that higher radiation dose could perform better outcomes for esophageal squamous cell carcinoma patients.

## INTRODUCTION

Esophageal cancer (EC) is a highly lethal malignancy over the world [1, 2]. In East Asia, esophageal squamous cell carcinoma (ESCC) is the most common type, whereas adenocarcinoma is predominant in Western countries. [3]. More than 50% of patients with EC when diagnosed are at late stages and not amendable at all to major surgery [4]. At present, definitive concurrent chemoradiotherapy (CCRT) is the common strategy for locally advanced inoperable EC patients based on the phase III intergroup trial RTOG 8501, which significantly improved the local control (LC) and overall survival (OS) compared with radiotherapy (RT) alone [5, 6]. In the landmark INT0123 trial, the results demonstrated that escalating the dose to 64.8 Gy did not confer to a benefit compared with conventional doses, which may lead to a higher incidence of treatment-related toxicity [7]. On the basis of those clinical trials, 50.4 Gy is recommened as the standard radiation dose for definitive treatment to EC. However, the outcomes for patients with EC treated by standard dose radical radiotherapy were still disappointing. Thus, the optimal radiation dose of definitive CCRT for EC remains in debate. Several studies were performed to investigate the potential benefit of high dose radiotherapy for EC. Notably, most of the previous study grouping conditions are greater than or less than a threshold dose, such as greater than 60 Gy or less than 60 Gy, greater than 50.4 Gy or less than 50.4 Gy. There is no study explored the efficacy of 59.4 Gy or higher radiotherapy compared with standard dose (50.4) radiotherapy on survival. Thus, we undertook the retrospective study to analyze the survival prognosis of patients who treated with different radiotherapy dose, and attempted to afford new evidences of choosing optimal radiation dose in EC patients treated with radical CRT.

## RESULTS

### Patient characteristics

A total of 137 patients with ESCC treated with CRT were identified, with a median follow-up of 27.5 months (6.4–79.5 months). Of these, 63 patients received standard doses, and 74 received high doses. The general characteristics of the enrolled patients were listed in Table 1. The complete response (CR), partial response (PR) and no-response (NR) rates in the standard-dose group were 39.7% (25/63), 36.5% (23/63) and 23.8% (15/63), respectively, while the rates were 48.6% (36/74), 36.5% (27/74) and 14.9% (11/74), respectively, in the high-dose group. The CR in the high-dose group was greater than that the standard-dose group (48.6% vs 39.7%, *P* = 0.03).

**Table 1:** Patient, disease, and treatment characteristics

Characteristics	Value or No.of Patients (%)	Low-Dose Group (≤ 50.4 Gy)	High-Dose Group (> 50.4 Gy)	*P* value
Age at diagnosis (yr)				
Median (range)	68 (36–81)	67 (38–79)	68 (36–81)	
Sex				
Male	95	45	50	0.625
Female	42	18	24	
Smoked at diagnosis				
No	55	24	31	0.651
Yes	82	39	43	
KPS				
90–100	86	39	47	0.846
≤ 80	51	24	27	
Tumor location				
Proximal	29	13	16	0.963
Middle	57	27	30	
Distal	51	23	28	
Tumor length (cm)				
Median (range)		5.0 (1.0–11.0)	5.0 (1.5–13.0)	
≤ 5	74	35	39	0.738
> 5	63	28	35	
Clinical T status				
T1	9	4	5	0.172
T2	37	18	19	
T3	59	27	32	
T4	32	14	18	
LN status				
N0	45	29	25	0.533
N1	63	31	32	
N2	26	14	12	
N3	3	2	1	
Clinical stage				
I	4	2	2	0.634
II	52	24	26	
III	81	41	40	
Chemotherapy				
Induced chemotherapy	24	13	11	0.811
Concurrent chemotherapy	65	31	34	
Sequential chemotherapy	14	6	8	
None	34	17	17	
Clinical response of primary tumor				
Complete response	61	25	36	0.359
Partial response	50	23	27	
None	26	15	11	
Second-line therapy				
adjuvant chemotherapy	95	49	46	0.394
Salvage esophagectomy	25	11	14	
Supportive treatment	17	8	9	
RT technique				
3D-CRT	79	38	41	0.631
IMRT	58	30	28	
PET examination				
Yes	23	10	13	0.578
No	114	56	58	

Abbreviations: KPS, karnofsky performance score; LN, lymph node; 3D-CRT, 3 dimensional-conformal radiotherapy; IMRT, intensity-modulated radiotherapy; RT, radiotherapy.

### Therapy outcomes

**Figure 1:**
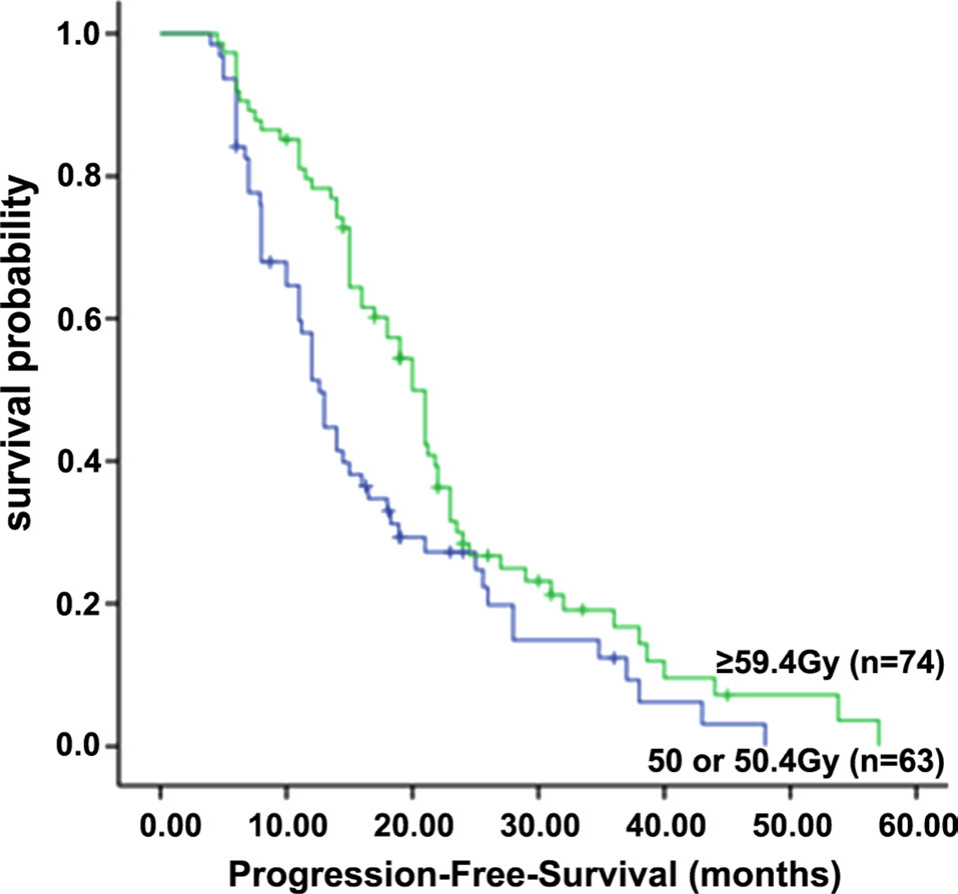
Progression-free survival of patients in the standard-dose group (50 or 50.4 Gy) and high-dose group (≥ 59.4 Gy)

For the entire cohort of patients, the 1-, 2-, and 3-year PFS rates were 70.0%, 29.1%, and 16.0%, respectively, with a median PFS of 16.5 months. As shown in Figure 1, the median PFS was 12.6 months, and the 1-, 2-, and 3-year PFS rates were 58.0%, 26.0% and 12.0%, respectively, in the standard-dose group; while in the high-dose group, the median PFS was 20.0 months, and the 1-, 2-, and 3-year PFS rates were 80.1%, 31.0% and 20.0%, respectively (*P* = 0.013).

**Figure 2:**
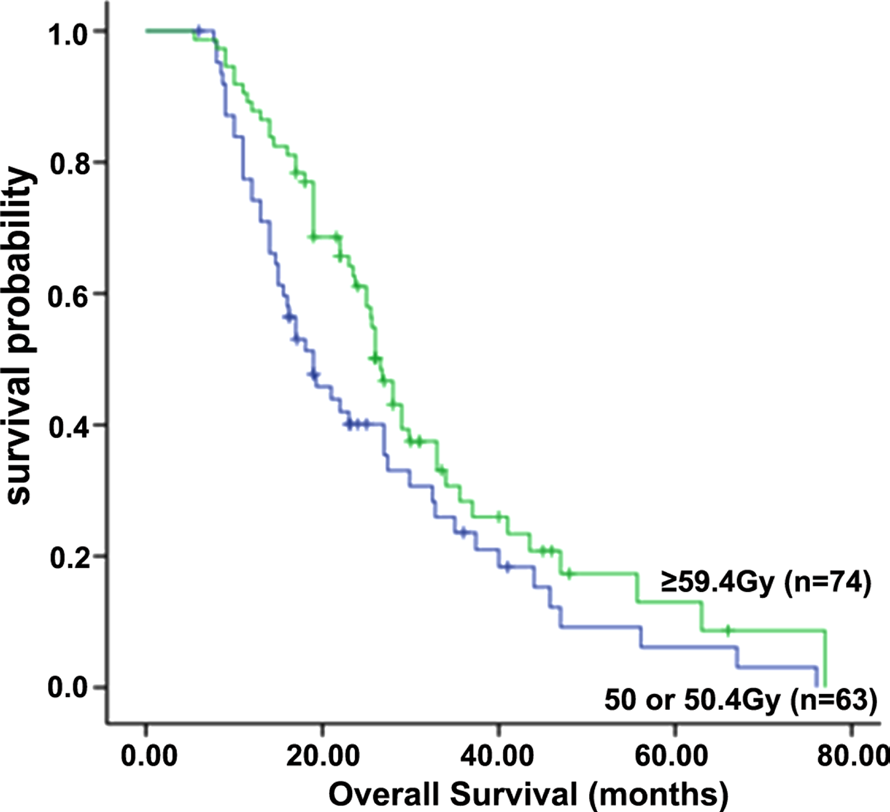
Overall survival of patients in the standard-dose group (50 or 50.4 Gy) and high-dose group (≥ 59.4 Gy)

For the entire cohort of patients, the 1-, 2-, and 3-year OS rates were 84.0%, 51.0%, and 27.2%, respectively, with a median OS of 25.0 months. The median OS was 19.0 months, and the 1-, 2- and 3-year survival rates were 78.0%, 39.0%, and 24.0%, respectively, in the standard-dose group; and, in the high-dose group, the median OS was 26.6 months, and the 1-, 2- and 3-year survival rates were 89.0%, 61.0%, and 30.0%, respectively (*P* = 0.037) (Figure 2). All endpoints were found to have statistically significant differences favoring the high-dose group.

### Toxicity

Radiation toxicities were mainly esophagitis, hematologic toxicity, and radiation pneumonia. The rate of grade ≥ 3 radiation esophagitis was much more frequent in the high-dose group (10.5% vs. 2.2% for the standard-dose group, *p* < 0.01). Grade ≥ 3 radiation pneumonitis occurred in 4% of patients in the standard-dose group and 6% of patients in the high-dose group, respectively. There was a trend to increase the treatment-related toxicity, but it was not statistically significant (*p* > 0.05). Furthermore, other toxicities did not differ significantly between groups. There were two treatment-related deaths in the standard-dose group (esophageal bleeding, one patient; radiation pneumonitis, one patient) and five treatment-related deaths in the high-dose group (aspiration pneumonia due to trachea-esophageal fistula in 1 to 3 months after 60 Gy, two patient; tumor bleeding in 3 months, three patients).

### Prognostic factors

Patient characteristics were evaluated to determine their prognostic value in the terms of OS (Table 2). According to a univariate analysis, Univariate analysis revealed that smoking status, karnofsky performance status, clinical T stage, and RT dose were found to be significant risk factor. Multivariate analysis revealed that clinical stage, RT dose ≥ 60 Gy, and karnofsky performance status were independent prognostic factors for OS.

**Table 2:** Univariate and multivariate analysis of prognostic factors for OS

Factors	Univariate analysis			Multivariate analysis		
	HR	95% CI	*P*	HR	95% CI	*P*
Age (≥ 60)	0.83	0.41–1.62	0.53			
Sex (Male)	0.683	0.39–1.27	0.226			
Smoking status (No)	0.52	0.25–0.93	0.037	1.143	0.74–1.72	0.532
KPS (≥ 90)	0.41	0.24–0.70	< 0.01	0.39	0.21–0.68	< 0.01
Tumor location (Distal)	0.63	0.33–1.19	0.14			
Tumor length (≤ 5 cm)	0.788	0.49–1.28	0.334			
Tumor stage (T1–T3)	0.691	0.48–0.96	0.045	0.663	0.45–0.94	0.031
LN status (No)	0.50	0.24–1.04	0.07			
RT dose (≥ 59.4)	0.43	0.25–0.81	0.01	0.40	0.23–0.77	< 0.01

## DISCUSSION

During the last three decades, major advances in surgery, RT and chemotherapy have established multimodal approaches as curative treatment options for EC. For patients with inoperable or irresectable diseases, definitive CCRT is the choice of treatment. The NCCN recommended EC radical radiotherapy dose is on the basis of the results of the pivotal study of INT0123 study [7]. However, the study has been criticized due to several drawbacks surround the interpretation of the results. Firstly, the survival between the two groups was influenced by a great deal of complicated deaths among patients in high radiation doses arm (11 *vs.* 2 deaths). In point of fact, this may not be due to radiation dose escalation as most of deaths in the high radiation doses group occurred before receiving a cumulative dose greater than 50 Gy. Obviously, most of the causes of deaths were related to chemotherapy. Furthermore, a significantly lower dose of 5-FU was administered to patients received radiation dose of 64.8Gy, which could negatively affect the outcomes of the high-dose arm. Another study conducted by Gaspar et al. also failed to improve the outcomes and resulted in an unacceptably high rate of radiotherapy related toxicity. The standard radiation dose for patients with inoperable EC receiving definitive chemoradiation therapy has remained 50.4 Gy [8]. Notably, a great deal of studies revealed that the most common recurrence pattern for esophageal cancer after definitive CRT is local-regional recurrence and that a majority of local recurrence occur in the gross tumor volume even for patients achieved complete remission after treatment [9–15]. It indicated that standard dose (50.4 Gy in 28 fractions) was not enough to obtaina higher local control rates.The recommendation of NCCN guidelines for radical radiation dose of EC was 50 or 50.4 Gy, but the optimal radiation dose of CRT for EC should be reevaluated.

Several studies have attempted to verify the benefit of radiation dose escalation in definitive CRT for locally advanced esophageal cancer. Zhang et al. investigated 69 unresectable or refused resection EC patients who underwent radical CRT, including 43 who received ≤ 51 Gy and 26 who received > 51 Gy [16]. They found that patients had a greater 3-year LC (36% *vs.* 19%, *p* = 0.011) and disease-free survival (DFS) (25% *vs.* 10%, *p* = 0.004) in the high radiation doses arm than those in the low dose group, but that OS was not significantly different (13% vs. 3%, *P* = 0.054). In addition, data from Norway also dose escalation could be beneficial for patients with EC, which could provide a better local control [17]. The OS for patients with higher radiation doseo f definitive CRT was, however, not better than patients receiving lower radiation dose of chemoradiotherapy. Wang et al. retrospectively assessed the outcomes of patients with M0 cervical and upper thoracic esophageal cancer from MD Anderson Cancer Center and demonstrated that patients who received > 50 Gy experienced greater levels of a CR than patients treated with < 50 Gy (79.2% vs 27.3%; *P* = 0.003) [18]. On multivariable analysis, the radiation dose was the only factor predictive of OS, with improvement in survival in the group receiving > 50 Gy. In the retrospective analysis by Suh YG and colleagues, the results also showed patients who received a total dose ≥ 50.4 Gy of RT had significantly better LRC (69% vs. 32%, *p* < 0.01) and PFS (47% vs. 20%, *p* = 0.01), than patients receiving < 50 Gy when treated with concurrentchemotherapy. High-dose radiation ≥ 50.4 Gy showed no significant OS benefit for patients with EC (28 vs. 18 months, *p* = 0.26) [19]. More recently, additional retrospective analyses from MD Anderson Cancer Center reported outcomes from a cohort of 193 patients with ESCC underwent radical concurrent chemotherapy and radiation therapy using modern techniques [20]. The results showed that high radiation dose provided a significant lower rate of local recurrence (17.9% vs 34.3%, *p* = 0.024) compared to patients receiving low radiation dose. Furthermore, patients receiving high radiation did have a marginal better 5-year local regional recurrence-free survival (68.7% vs 55.9%, *p* = 0.052) than low-dose group. The 5-year overall survival rate was no significant differences between the two groups (*p* = 0.617).It revealed that high radiation doses improved tumor local control but could not bring a survival benefit for patients with ESCC.A retrospective analysis performed by Kim et al was conducted to investigate the outcomes that high radiation did canbring a survival benefit for EC patients treated with CRT [21]. The results showed that patients receiving high radiation dose had a greater 2-year LRC (69.1% vs. 50.3%, *p* = 0.002), median PFS (16.7 vs. 11.7 months, *p* = 0.029), and median survival time (35.1 vs. 22.3 months, *p* = 0.043) than the low-dose group. The optimal radiation dose of radical CRT for EC, however, is still not definite (as summaried in Table 3). These conflicting results could be potentially attributed to differences in patient populations, tumor histology types, grouping criteria, as well as treatment.

**Table 3:** High-dose versus standard-dose radiotherapy for ESCC

References	Radiation dose	Radiation technique	*n*	LC	*P* value	OS	*P* value
Minsky et al.[7]	50.4 Gy	Conventional RT	109	46%	> 0.05	40% (2 year)	> 0.05
	64.8 Gy		109	48%		31%	
Zhang et al. [16]	< 51 Gy	Conventional RT	43	19%	0.011	3% (3year)	0.054
	≥ 51 Gy		26	36%		13%	
Hurmuzlu et al. [17]	≥ 60 Gy	Conventional RT	46	33 months (MST)	–	22% (2 year)	–
Wang et al. [18]	< 50 Gy	Conventional RT	11	47.7% (whole	0.001	0% (5 year)	0.002
	> 50 Gy	or 3D-CRT	24	group)		29%	
Suh et al. [19]	< 60 Gy	Conventional RT	49	32%	< 0.01	18 mons (MST)	0.26
	≥ 60 Gy		77	69%		28 mons	
He et al. [20]	≤ 50.4 Gy	3D-CRT	137	34.3% (LFR)	0.024	33.0% (5 year)	0.617
	> 50.4 Gy		56	17.9%		41.7%	
Kim et al. [21]	< 60 Gy	3D-RT or IMRT	120	37.3%	0.02	22.3 mons (MST)	0.043
	≥ 60 Gy		116	59.7%		35.1 mons	

Abbreviations: LC, local control; RT, radiotherapy; MST, median survival time; OS, overall survival; LRF, Local failure rate.

In our study, homogenous histologic type of ESCC was included and all patients received similarly chemotherapy regimens. Our study showed that higher radiation dose brings a survival benefit to stage II–III ESCC patients treated with definitive CRT. The results revealed that both progression free survival and median survival in the high radiation dose arm are longer than that in the conventional radiation dose arm. The 1-, 2-, and 3-year PFS rates in the high-dose group were higher in high radiation dose arm than that in the standard-dose arm. It is the same to the median OS. With regard to toxicity, the rate of grade ≥ 3 acute and late radiation related toxicity seem to be high in the landmark INT0123 trial. Recently, several studies used modern radiotherapy technique have report that the rate of grade ≥ 3 radiation esophagitis and radiation pneumonitis were about 1 0.8%–18% and 3.3%–6% in EC patients treated with CRT [16–21]. In our study, the incidence of grade 3 or higher radiation esophagitis was higher in the high radiation dose arm than that in the standard radiation dose arm (10.5 vs.2.2%, *p* < 0.01). The incidence of grade greater than or equal to 3 radiation pneumonitis during and after completing the treatment were 4% in the group received a standard dose (50.4 Gy) and 6% in the group received a higher dose of RT(≥ 59.4 Gy). In addition, there was no significant difference between the two groups in terms of other treatment-related toxicities. The Adverse event rates seem to be low in our study. This may explained by that the radiation technique used in previous studies were two-dimensional, and the margins applied to the target volume were larger than those used in current practice, which may have increased the probability of toxicities. However, in our study, the application of precision radiotherapy and simultaneous modulated accelerated radiotherapy, which can reduce incidences of treatment toxicities. Moreover, nearly half of the patients received involved-field radiotherapy (IFRT), and studies showed that IFRT lead to reduce treatment toxicities without sacrificing overall survival in patients with EC [22–25]. In addition, the follow-up time of some patients in the current study was not long enough. Observation period is not sufficient for evaluating the overall effects of CRT. The results of our study indicated that patients who received radiation doses in excess of 59.4 Gy would have significantly better PFS, and OS than patients receiving standard dose (50–50.4 Gy) when treated with concurrent chemotherapy, which are similar to the results of Kim and colleague [21].

There are limitations to our approach in this study. First, it was a retrospective study, and our data originated from a single institution. Furthermore, the data of radiation planning parameters, including field size, which may have influenced the outcomes. Finally, it is possible that treatment-related toxicities were underestimated due to the study’s retrospective setting.

## MATERIALS AND METHODS

### Patient characteristics

We retrospectively reviewed non-operated localized ESCC patients who received CCRT with external beam radiotherapy and diagnosed within 2010–2014. All patients had histologically proven primary ESCC. Between May 2010 and May 2014, a total of 366 patients with ESCC were treated by CCRT at our institutions. Patients were excluded from this analysis for the following reasons: (1) patients received radiotherapy with palliative intent (*n* = 78); (2) they underwent esophagectomy after CRT (*n* = 46); (3) patients did not receive complete treatment (*n* = 49); (4) follow-up loss after CRT (*n* = 44) and (5) they had prior malignancies (*n* = 22). Ultimately, the medical records of total 137 patients treated with CCRT were retrospectively reviewed for this study. The electronic medical records of those patients were retrospectively reviewed. Pretreatment investigations included medical history, physical examination, symptoms, performance status, complete blood count, measurement of serum electrolytes, CT of the neck, chest and abdomen, bone scan, endoscopy, and esophagography. Part of patients underwent 18F-fluorodeoxyglucose positron emission tomography (FDG-PET). The clinical stage was assigned according to the 7th American Joint Committee on Cancer (AJCC) staging system. Patients were stratified by the total radiation dose, with the high-dose group receiving ≥ 59.4 Gy and the standard dose group by 50 or 50.4 Gy.

### Treatment approaches

All patients underwent radiotherapy delivered by three-dimensional conformal radiation therapy or intensity-modulated radiation therapy technique. Patients were treated 5 days per week at 1.8–2.0 Gy. The gross tumor volume (GTV) was delineated based on CT, barium esophagogram, endoscopic examination, and PET imaging. The clinical target volume (CTV) consisted of clinical target volume (CTVt) (the gross tumor volume) and nodal CTV. The CTVt included the gross primary tumor volume with a radial margin of 0.5 to 1 cm and a proximal and distal margin of 3 to 5 cm. The nodal CTV was defined by a 0.5 to 0.8 cm expansion around the nodal gross tumor volume and some patients also covered the regional nodal regions. The planning target volume (PTV) was the CTV plus a uniform 0.5-cm expansion margin. For both the standard-dose group (50 or 50.4 Gy) and high-dose (≥ 59.4 Gy) groups, prescribed dose is given to the PTV. Chemotherapy consisted of two cycles of platinum-based chemotherapy combined with 5-fluorouracil and a taxane (docetaxel or paclitaxel). In the whole group, 103 (75.2%) patients underwent chemotherapy, including induction chemotherapy (23.3%), concurrent chemotherapy (63.1%), and sequential chemotherapy (13.6%). Among the patients who underwent chemotherapy, 53 (51.5%) were in the high-dose group and 50 (48.5%) were in the standard-dose group.

### Assessment of response and toxicity

Tumor response was evaluated according to the Response Evaluation Criteria in Solid Tumors (RECIST) system [26]. Treatment-related toxicities were recorded according to the common toxicity criteria for adverse events (version 3.0) [27]. The observation started from the date of treatment to the date of death or the last follow-up.

### Follow-up

All patients were examined weekly during RT to monitor treatment toxicities and their general condition. Routine evaluations included physical exam, hematologic and biochemical profiles, and esophagography. The patients were evaluated at 1 month after RT. Thereafter, the patients were asked to visit our clinic at 3-month intervals for the first 2 years, and then at every 6 months’ interval. The follow-up evaluations included physical examination, esophagography, esophagogastroduodenoscopy, chest and abdominal CT and positron emission tomography (PET) when available. Other necessary examinations were conducted according to the clinical situation.

### Definition of event

OS was measured from the first day of treatment to the date of death from any cause or the last known date that the patient was alive. PFS was defined as the duration from the date of treatment to the date of failure, either the date of death from any cause or the date of the last known follow-up.

### Statistical analysis

Statistical analyses were carried out using SPSS (version 19.0). Comparisons of patient characteristics and toxicities were performed with Chi-square (and Fisher’s exact) test. OS and PFS were calculated using the Kaplan-Meier method, and the log-rank test was employed to evaluate the difference in survival curves between the two groups. Multivariate analysis for OS was performed using Cox regression. Statistical tests were based on a two-sided significance level, and *p* values of less than 0.05 were considered significant.

## CONCLUSIONS

In summary, the results of our study revealed that stage II-III patients who delivery of radiation dose above the current standard 50.4 Gy could prolong survival time, including PFS and OS. Although modern radiation techniques could ensure the safety and effectiveness of delivering higher radiation doses, controversies still exist about the optimal radiation dose for patients with ESCC undergoing radical treatment. Thus, in the future, randomized prospective clinical studies are needed to verify the effect of radiation dose escalation of CCRT for EC.
